# Intranasal delivery of a subunit protein vaccine provides protective immunity against JN.1 and XBB-lineage variants

**DOI:** 10.1038/s41392-024-02025-6

**Published:** 2024-11-20

**Authors:** Hong Lei, Weiqi Hong, Jingyun Yang, Cai He, Yanan Zhou, Yu Zhang, Aqu Alu, Jie Shi, Jian Liu, Furong qin, Danyi Ao, Xiya Huang, Zimin Chen, Hao Yang, Yun Yang, Wenhai Yu, Cong Tang, Junbin Wang, Bai Li, Qing Huang, Hongbo Hu, Wei Cheng, Haohao Dong, Jian Lei, Lu Chen, Xikun Zhou, Jiong Li, Li Yang, Zhenling Wang, Wei Wang, Guobo Shen, Jinliang Yang, Zhiwei Zhao, Xiangrong Song, Guangwen Lu, Qiangming Sun, Youchun Wang, Shuaiyao Lu, Xiawei Wei

**Affiliations:** 1 Laboratory of Aging Research and Cancer Drug Target, State Key Laboratory of Biotherapy and Cancer Center, National Clinical Research Center for Geriatrics, West China Hospital, Sichuan University, No. 17, Block 3, Southern Renmin Road, Chengdu, Sichuan 610041 PR China; 2 National Kunming High-level Biosafety Primate Research Center, Institute of Medical Biology, Chinese Academy of Medical Sciences and Peking Union Medical College, Yunnan, China

**Keywords:** Vaccines, Infectious diseases

## Abstract

The mucosal immune response plays a crucial role in the prevention of respiratory viruses. Given the risk of recurrent SARS-CoV-2 infections in the population, the rapid development of next-generation intranasal COVID-19 vaccines with high safety and efficacy is paramount. In the current study, we developed a protein-based intranasal vaccine comprising the XBB.1.5 receptor binding domain (RBD)-derived trimeric recombinant protein (RBD_XBB.1.5_-HR) and an MF59-like oil-in-water adjuvant. Intranasal administration of RBD_XBB.1.5_-HR vaccine elicited robust and sustained humoral immune responses in mice and rats, resulting in high levels of neutralizing antibodies against XBB-lineage subvariants, with protection lasting for at least six months. The intranasal RBD_XBB.1.5_-HR vaccine generated potent mucosal immune responses, characterized by the inductions of tissue-resident T (T_RM_) cells, local cellular immunity, germinal center, and memory B cell responses in the respiratory tract. The combination of intramuscular and intranasal delivery of the RBD_XBB.1.5_-HR vaccine demonstrated exceptional systemic and mucosal protective immunity. Furthermore, intranasal delivery of RBD_XBB.1.5_-HR vaccine as a heterologous booster shot showed more effective boosting effects after mRNA administration compared to homologous vaccination, as evidenced by the induction of superior systemic and extra mucosal immune response. Importantly, the intranasal RBD_XBB.1.5_-HR vaccine conferred efficient protection against the challenge with authentic EG.5.1 viruses in vivo. These findings identify the intranasal RBD_XBB.1.5_-HR vaccine as a potential mucosal vaccine candidate for the prevention of SARS-CoV-2 infection.

## Introduction

Since its emergence in January 2020, SARS-CoV-2, the virus responsible for the COVID-19 pandemic, has continuously evolved and spread worldwide. The detection of the Omicron variant (B.1.1.529) in November 2021, characterized by over 30 amino acid mutations, represented a major evolutionary shift. Omicron quickly became dominant, leading to several subvariants such as BA.2, BA.5, and BQ.1.1, which caused widespread infections. By early 2023, recombinant XBB lineages with enhanced immune evasion emerged, including subvariants like XBB.1.5, XBB.1.16 (F486P), EG.5 (with F456L mutation), and EG.5.1 (with F456L and Q52H mutations).^1–6^ Recently, the BA.2.86-derived subvariant JN.1,^7,8^ with an additional L455S mutation, has become the most reported variant of interest (VOI) globally, representing 25.7% of sequences. As of late July 2024, the latest dominant variants are KP.2 and KP.3, both descendants of JN.1, with KP.3 increasing to 29.4% and KP.2 to 12.8% of sequences, according to the World Health Organization.

Unlike the majority of currently approved COVID-19 vaccines administered via intramuscular injection, intranasal delivery of antigens presents a more promising approach to effectively halt SARS-CoV-2 shedding and transmission. This strategy capitalizes on the mechanism of action for intranasal vaccines involves the induction of mucosal immunity in addition to systemic immunity against SARS-CoV-2 at the site of viral entry. The mucosal response is characterized by the generation of mucosal secretory IgA (sIgA) antibodies and the induction of resident memory T (T_RM_) cells post-vaccination,^9–12^ which play a key role in preventing pathogens from entering the body through mucosal surfaces. Leveraging the advantages of activating mucosal immune responses, significant efforts have been dedicated to the development of intranasal vaccines.

The most utilized platforms for intranasal vaccine development are viral vector-based^10,12–14^ and subunit protein vaccines. However, the efficacy of viral vector vaccines can be compromised by pre-existing immunity from previous administrations. On the other hand, subunit protein vaccines are favored for their safety, cost-effectiveness in mass production, and ease of transportation. However, pure proteins are quickly cleared from the mucosa, leading to weak immune responses. To address this challenge, protein antigens have been adjuvanted with various intranasal adjuvant, including polymersomes,^15^ membrane vesicles,^16^ nanoparticles,^17^ and agonists of Toll-like receptors,^18,19^ to improve both the magnitude and durability of immune responses. In our prior research, we developed cationic crosslinked carbon dots (CCD) to augment the immunogenicity of proteins in the mucosal environment.^20^ However, these adjuvants have not yet achieved widespread clinical approval due to potential safety concerns. Therefore, selecting an intranasal adjuvant that has undergone clinical evaluation could significantly facilitate the clinical translation process.

MF59 is a licensed oil-in-water emulsion adjuvant effectively used in several influenza vaccines, including seasonal flu and pandemic H1N1 vaccines, known for its safety and efficacy in humans.^21–24^ Typically used in injectable vaccines, MF59 forms a depot at the injection site, enhancing antigen release and uptake. It recruits and activates antigen-presenting cells, boosts cytokine production, and enhances antibody and memory cell generation.^21,25^ In addition to its use in intramuscular vaccines, there is emerging interest in its application for intranasal vaccines. Several studies have shown that intranasal delivery of MF59-adjuvanted subunit influenza vaccines significantly enhances both mucosal and systemic immunogenicity in animal models,^26,27^ suggesting it holds promise for developing effective intranasal vaccines against SARS-CoV-2 infections.

Previously, we used the self-assembly properties of spike heptad-repeat (HR) sequences to trimerize the receptor binding domain (RBD)^28^ and updated the variant sequences to produce the trimeric protein RBD_XBB.1.5_-HR. To investigate whether the MF59-like adjuvanted RBD_XBB.1.5_-HR vaccine can be delivered intranasally and effectively serve as an intranasal COVID-19 vaccine remains uncertain. In this study, we aimed to evaluate the humoral and cellular immune responses induced by intranasal administration of the MF59-like oil-in-water adjuvant-formulated RBD_XBB.1.5_-HR vaccine both as a standalone vaccine and as a heterologous booster following mRNA vaccine injection. We also explored the potential of combining intramuscular and intranasal delivery to elicit a superior immune response. Additionally, we assessed the protective efficacy of the intranasal RBD_XBB.1.5_-HR vaccine against challenge with live EG.5.1 virus. This study seeks to examine the effectiveness of the intranasally adjuvanted RBD_XBB.1.5_-HR vaccine, and expedite the development and clinical translation process of next-generation intranasal COVID-19 vaccine.

## Results

### Intranasal delivery of adjuvanted RBD_XBB.1.5_-HR vaccine elicits cross-neutralization activities against JN.1 and XBB-lineages variants

**Fig. 1:**
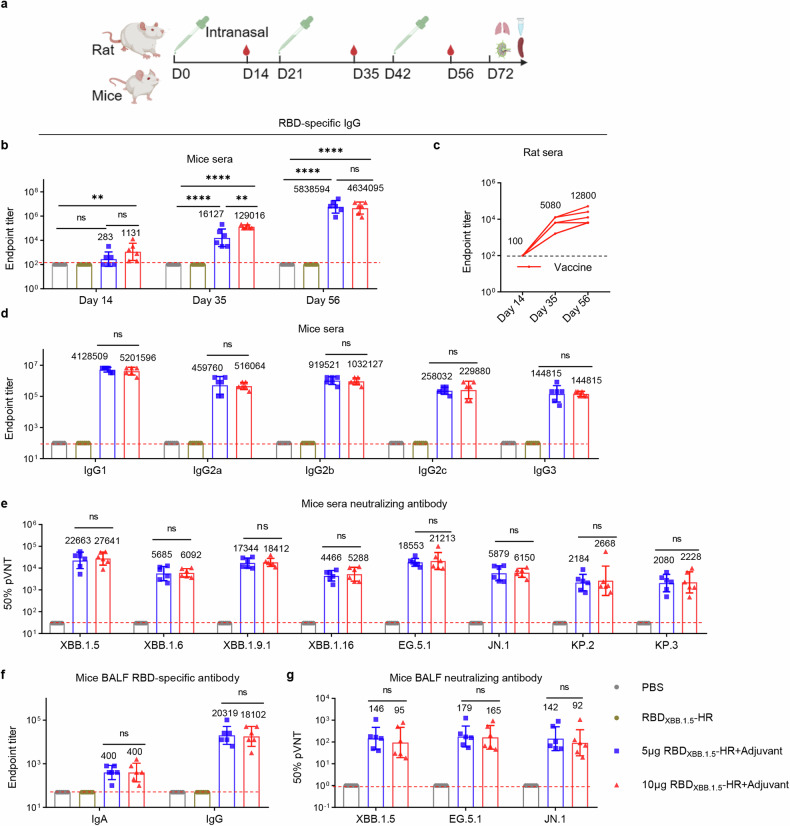
Intranasal delivery of adjuvanted RBD_XBB.1.5_-HR vaccine elicits strong humoral immune response with great levels of neutralizing antibodies. **a** The schematic representation of the immunization and sera collection protocol in mice and rats. The animals were intranasally administrated with adjuvanted RBD_XBB.1.5_-HR on days 0, 21 and 42, and the sera were collected on day 14 after each immunization. **b** Endpoint titers of anti-RBD IgG in sera from mice intranasally immunized with low dose (5 μg) and high dose (10 μg) of adjuvanted RBD_XBB.1.5_-HR vaccine (*n* = 6 mice per group). **c** Endpoint titers of sera anti-RBD IgG in rats received 40 μg of intranasal RBD_XBB.1.5_-HR vaccine (*n* = 5 rats per group). **d** Antibody subtypes of anti-RBD IgG in mouse sera collected on day 56 (*n* = 6 mice per group). **e** Neutralizing antibodies against pseudoviruses in mouse sera collected on day 56 (*n* = 6 mice per group). Endpoint titers of anti-RBD IgA, IgG (**f**) and neutralizing antibodies (**g**) in mouse bronchoalveolar lavage fluid (BALF) sample that collected on day 72 (*n* = 6 mice per group). Data are presented as geometric mean values ± SD in **b**, and **d–f**, and presented as geometric mean with individual value in **c**. *P* values were conducted by One-way ANOVA analysis followed by Tukey’s multiple comparisons test in **b**, and **d**–**g**. ****P < 0.0001; ***P < 0.001; **P < 0.01; *P < 0.05; ns not significant

We and others have reported that intranasal delivery of recombinant protein antigens alone, with poor immunogenicity, hardly induces visible immune responses. Therefore, our initial objective was to investigate whether the MF59-like oil-in-water adjuvant could enhance the antigenicity of the RBD_XBB.1.5_-HR protein in respiratory mucosa to induce substantial protective immunity. Mice were intranasally administrated with dose of 5 μg (low) or 10 μg (high) of adjuvanted-RBD_XBB.1.5_-HR protein vaccines following a prime-boost regimen with a 21-day interval (Fig. 1a). The mice received PBS or naked RBD_XBB.1.5_-HR were used as control groups. The antigen-specific binding antibody assay revealed that the RBD_XBB.1.5_-HR protein alone can hardly elicit sera (Fig. 1b) and mucosal (Fig. 1f) RBD-specific antibodies, whereas all animals in groups receiving immunization with adjuvanted proteins showed a significant improvement in the endpoint titers of antigen-specific IgG and IgA. Similar improvements were observed in rats with intranasal delivery of RBD_XBB.1.5_-HR vaccines (Fig. 1c). In addition, we found both Th1- and Th2-biased immune responses could be elicited by adjuvanted-RBD_XBB.1.5_-HR vaccine, manifested by the production of various antibody subtypes, including antigen-specific IgG1, IgG2a, IgG2b, IgG2c, and IgG3 antibodies (Fig. 1d).

The neutralizing antibody response serves as a crucial indicator of vaccine protective efficacy. Therefore, we subsequently assessed the neutralizing activities against circulating JN.1 and XBB-lineage variants following intranasal immunization. Although the levels of neutralizing antibodies (nAbs) showed only a modest improvement on day 35 after immunization (Supplementary Fig. 1), both doses resulted in orders of magnitude increase in neutralizing antibodies after the third dose (Fig. 1e). To be specific, the group receiving the low dose of adjuvanted-RBD_XBB.1.5_-HR vaccination exhibited geometric mean titers (GMTs) for 50% neutralization against various pseudoviruses, including XBB.1.5 (22,663), XBB.1.6 (5,685), XBB.1.9.1 (17,344), XBB.1.16 (4,466), and EG.5.1 (18,553). In contrast, the GMTs for the high dose group were 27,641, 6,092, 18,412, 5,288, and 21,213, respectively (Fig. 1e). Notably, the emerging subvariant JN.1, a descendant of BA.2.86, along with its lineages KP.2 and KP.3, has raised concerns regarding potential immune escape. Although the neutralizing activities induced by the adjuvanted-RBD_XBB.1.5_-HR vaccination were reduced against these subvariants compared to XBB lineages, GMTs against JN.1 were 5,879 and 6,150 for the low- dose and high-dose groups, respectively, and both KP.2 and KP.3 had GMTs above 2,000, suggesting that the vaccine still offers a certain level of protection against these circulating variants.

Furthermore, besides the serum samples, the adjuvanted-RBD_XBB.1.5_-HR vaccination elicited a neutralization response in the local respiratory mucosa, as evidenced by elevated neutralizing antibody levels in bronchoalveolar lavage fluids (BALF) (Fig. 1f, g). This highlights that intranasal delivery of the adjuvanted-RBD_XBB.1.5_-HR vaccine can induce both systemic and mucosal humoral immune responses, characterized by high levels of neutralizing antibodies against both JN.1 and XBB-lineage variants.

Establishing a sustained antibody response is crucial for protection against the ongoing COVID-19 pandemic. To address this, we assessed binding antibodies (Supplementary Fig. 2a) and neutralizing activities (Supplementary Fig. 2b) at extended intervals following the final intranasal immunization with RBD_XBB.1.5_-HR. Although antibody levels showed a decline compared to day 56, the pseudovirus neutralization assay demonstrated that neutralizing antibodies against various variants were maintained six months after the completion of immunization (Supplementary Fig. 2b).

### Intranasal delivery of adjuvanted RBD_XBB.1.5_-HR vaccine induces robust airway cellular immune response

**Fig. 2:**
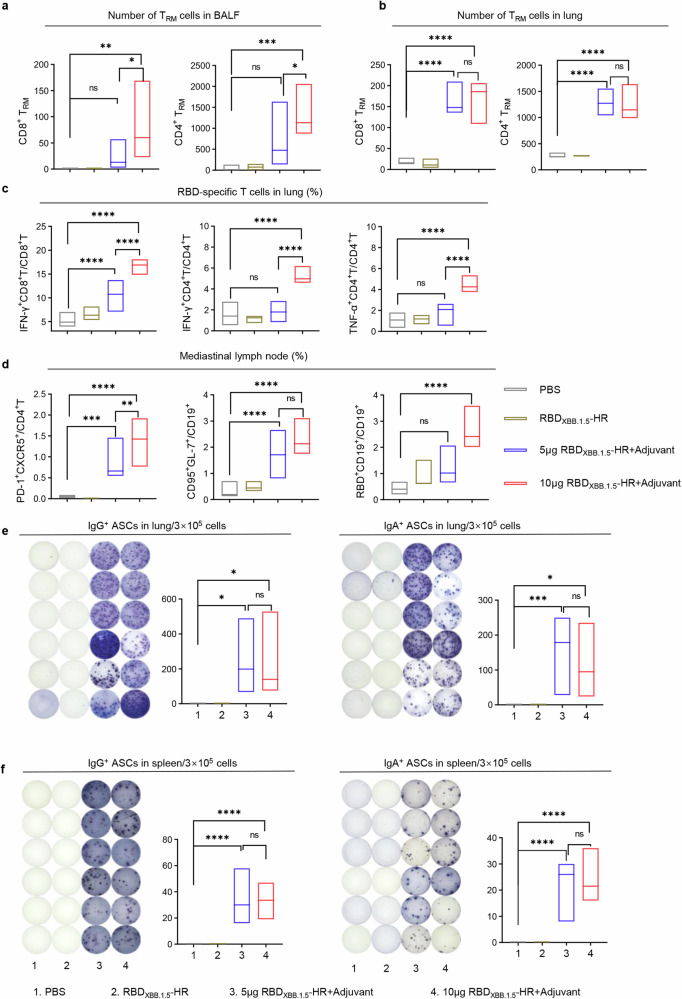
Intranasal delivery of adjuvanted RBD_XBB.1.5_-HR vaccine induces strong local mucosal immune response. The absolute number of CD8^+^ and CD4^+^ tissue-resident memory T (T_RM_) cells in (**a**) BALF and (**b**) lung samples from vaccinated mice. T_RM_ cells were gated on CD44^+^CD62L^-^CD69^+^CD4^+^ or CD44^+^CD69^+^CD103^+^CD8^+^ (*n* = 5 mice each group). **c** The percentages of antigen specific IFN-γ or TNF-α-producing memory CD8^+^ and CD4^+^ T cells in lung tissue after stimulation with peptide pools for SARS-CoV-2 XBB.1.5 spike (*n* = 6 mice each group). **d** The frequencies of T follicular helper cells (CD4^+^PD-1^+^CXCR5^+^), germinal center B cells (CD19^+^GL7^+^CD95^+^), and RBD-specific B cells (RBD^+^CD19^+^) in mediastinal lymph nodes (*n* = 6 mice each group). The representative images and quantitative analysis of RBD-specific IgG (left) and IgA (right) antibody secreting cells (ASCs) in the (**e**) lung and (**f**) spleen tissues (*n* = 6 mice per group). The middle line indicates the median and the box shows the data range. *P* values were conducted by One-way ANOVA analysis followed by Tukey’s multiple comparisons test in **a–f**. ****P < 0.0001; ***P < 0.001; **P < 0.01; *P < 0.05; ns not significant

Tissue-resident memory (T_RM_) cells are considered essential components of the mucosal immune response in host defense, as they can promptly respond to pathogens at the site of infection. We subsequently quantified the total count of CD8^+^ and CD4^+^ T_RM_s in BALF and lung tissues from immunized mice. Both doses of the intranasal adjuvanted-RBD_XBB.1.5_-HR vaccine led to a substantial increase in the number of T_RM_ cells in the respiratory tract (Fig. 2a, b). While there was no statistically significant difference in the number of T_RM_ cells in BALF samples between the control group and the low-dose vaccine group, an increase in frequencies was observed.

The lung antigen-specific cellular immune response was further evaluated employing intracellular cytokines staining (ICS). Pulmonary tissues from vaccinated mice were isolated and processed into a single-cell suspension. Subsequently, ex vivo stimulation was performed using full-length spike peptide pools to assess intracellular cytokine expression. T cells in the lungs exhibited a resting state in the absence of stimulation of peptide pools (Supplementary Fig. 3), while we noticed that both doses of intranasal RBD_XBB.1.5_-HR vaccine led to significant increases in the percentages of IFN-γ-secreting CD8^+^ T cells, as well as IFN-γ- and TNF-α-secreting CD4^+^ T cells after stimulation, with high-dose immunization further enhancing these improvements (Fig. 2c). Therefore, the induction of a variety of cytokine secretions in antigen-specific T cells suggests a multifunctional cellular immune response in the respiratory mucosa.

Germinal center (GC) responses induced by the intranasal RBD_XBB.1.5_-HR vaccine in mediastinal lymph nodes (mLN) were also assessed, since GC B and T follicular helper (Tfh) cells play a vital role in sustaining the long-term protective immune response. As anticipated, the proportions of GC B (CD19^+^GL7^+^CD95^+^) and Tfh (CD4^+^CXCR5^+^PD-1^+^) cells were significantly higher in immunized mice, compared to those receiving PBS and naked RBD_XBB.1.5_-HR (Fig. 2d). In addition, a greater number of RBD-specific B cells were detected in the mLN, indicating an increased presence of cells involved in antibody production (Fig. 2d). Enzyme-Linked ImmunoSpot (ELISpot) assays were then performed to measure the number of antibody-secreting cells (ASCs) in lung and spleen tissues. As expected, both doses of the intranasal vaccine induced the production of IgG- and IgA-ASCs in both local mucosal and systemic tissues, as evidenced by spot formation in cell samples from the immunized groups following incubation with pre-coated RBD_XBB.1.5_ -HR proteins (Fig. 2e, f).

In addition to the long-lasting antibody response (Supplementary Fig. 2a, b), we also evaluated memory B cells (MBCs) in the mucosal and spleen tissues, which are crucial for sustaining long-term antibody production.^29,30^ Significant increases in the percentages of MBCs were observed in the lung and mLN tissues, but not in the spleen, of mice receiving the intranasal vaccine nine months after the final dose (Supplementary Fig. 2c). This lack of change in the spleen could be attributed to the relatively weak systemic immune response induced by intranasal delivery. Nonetheless, these findings strongly support the idea that the intranasal RBD_XBB.1.5_-HR vaccine effectively elicits a robust and durable mucosal immune response.

### The combination of intramuscular and intranasal delivery of RBD_XBB.1.5_-HR vaccine elicits superior protective immunity

**Fig. 3:**
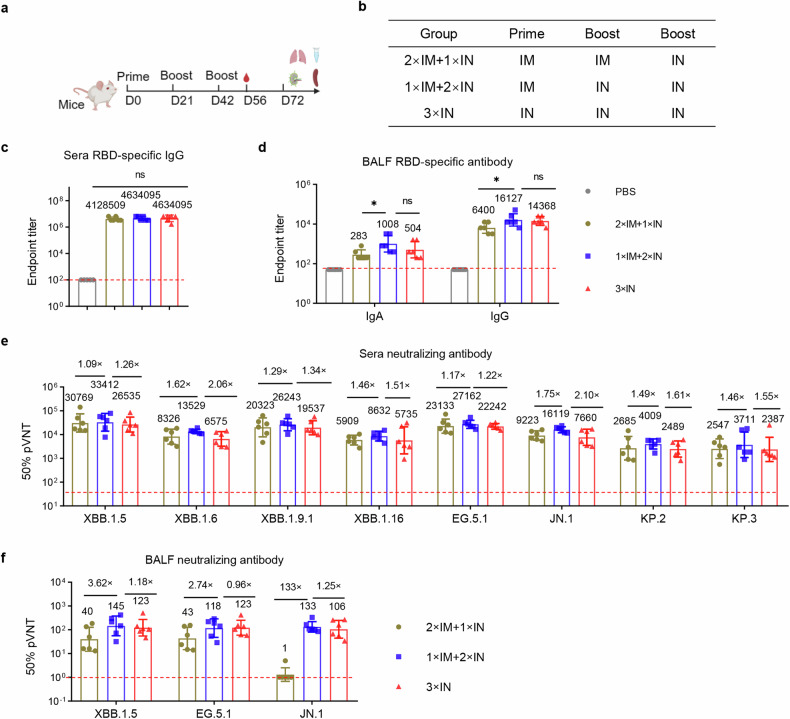
Combination of intramuscular and intranasal immunization elicits superior mucosal and systemic humoral immune response. **a**, **b** NIH mice were intramuscularly injected two doses of RBD_XBB.1.5_-HR vaccine, followed by one intranasal delivery (2×IM + 1×IN), or injected one dose of RBD_XBB.1.5_-HR followed by twice intranasal delivery (1×IM + 2×IN). Mice received three intranasal deliveries of intranasal RBD_XBB.1.5_-HR vaccine (3×IN) were used as control (*n* = 6 mice per group). Endpoint titers of RBD-specific IgG in sera (**c**), and IgA and IgG in BALF samples (**d**). The neutralization against XBB and JN.1-lineage pseudoviruses in sera (**e**) and BALF samples (**f**). Data are presented as geometric mean values ± SD in **c–f**. *P* values were conducted by One-way ANOVA analysis followed by Tukey’s multiple comparison post hoc test in **c**, **d**. ****P < 0.0001; ***P < 0.001; **P < 0.01; *P < 0.05; ns not significant

To further explore the potential of combining intramuscular and intranasal delivery of the RBD_XBB.1.5_-HR vaccine and determine whether this combined approach can elicit a superior immune response, we conducted immunization experiments in mice using different vaccination regimens (Fig. 3a, b). National Institute of Health (NIH) Swiss mice were initially given intramuscular injections of the RBD_XBB.1.5_-HR vaccine on days 0 and 21, followed by a third dose administered intranasally on day 42 (2×IM + 1×IN). Additionally, mice were intramuscularly administered the vaccine and subsequently received two booster doses through intranasal administration (1×IM + 2×IN). Reference groups consisted of mice treated with PBS or three doses of the intranasal RBD_XBB.1.5_-HR vaccine administered via intranasal delivery (3×IN).

There were no differences observed in serum RBD-specific IgG antibody levels among the three vaccination regimens (Fig. 3c). However, notably, the 1×IM + 2×IN immunization regimen produced the highest neutralizing antibodies levels among all groups tested (Fig. 3e). While all regimens involving at least one intranasal vaccine delivery induced significant antibody responses in the respiratory tract, the 1×IM + 2×IN regimen resulted in stronger binding and neutralizing antibody responses compared to the 2×IM + 1×IN group (Fig. 3d, f).

**Fig. 4:**
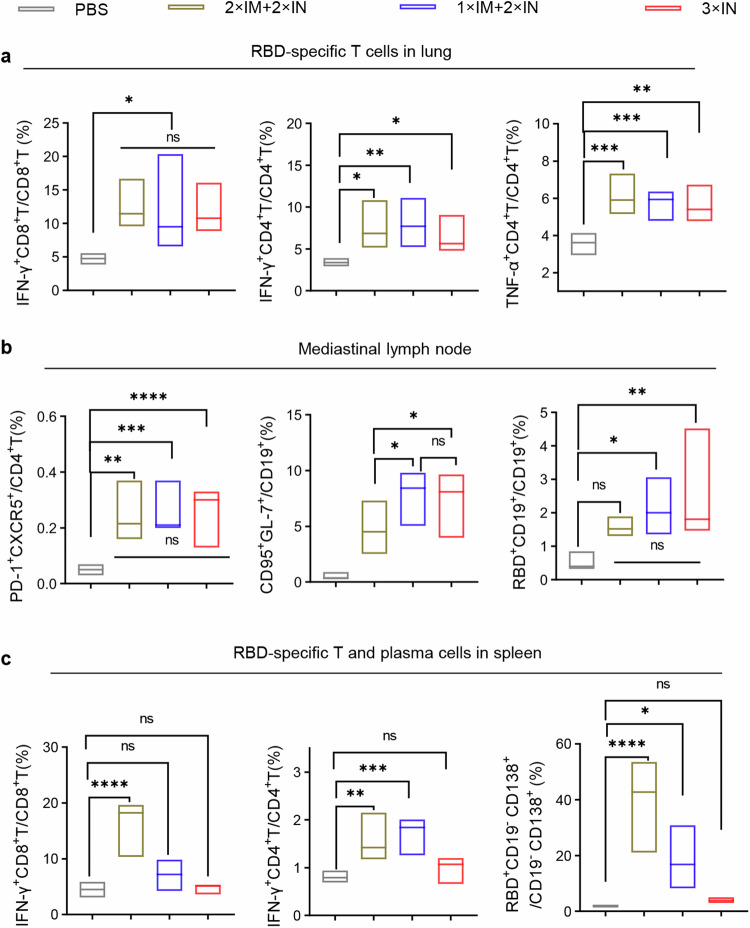
Combination of intramuscular and intranasal immunization elicits superior mucosal and systemic cellular immune response. **a** The percentages of antigen-specific IFN-γ- or TNF-α-producing memory T cells in lung tissue after stimulation with peptide pools of XBB.1.5 spike protein (*n* = 5 mice each group). **b** The frequencies of Tfh, GC B (CD19^+^GL7^+^CD95^+^), and antigen-specific B cells in mediastinal lymph nodes (*n* = 6 mice in each group). **c** The percentages of antigen-specific T cells and antigen-specific plasma cells (RBD^+^CD19^-^CD138^+^) in spleen tissue (*n* = 5 mice each group). The middle line indicates the median and the box shows the data range. *P* values were conducted by One-way ANOVA analysis followed by Tukey’s multiple comparison post hoc test. ****P < 0.0001; ***P < 0.001; **P < 0.01; *P < 0.05; ns not significant

To comprehensively assess the systemic and mucosal cellular immune responses induced by different vaccination regimens, we analyzed the generation of effector immune responses in spleen, lung, and lymph node tissues. All regimens that included at least one intranasal protein vaccine delivery demonstrated the ability to elicit substantial respiratory mucosal cellular immune responses (Fig. 4a). Furthermore, intranasal immunization induced germinal center responses in mediastinal lymph nodes, as evidenced by increased percentages of Tfh, GC B, and RBD-specific B cells (Fig. 4b). However, three intranasal deliveries of RBD_XBB.1.5_-HR vaccine resulted in negligible generation of antigen-specific T lymphocyte cells and plasma cells in splenic tissues, consistent with previous reports indicating that intranasal immunization elicits weak systemic immune responses (Fig. 4c, Supplementary Figs. 4–5). Importantly, the 2×IM + 1×IN or 1×IM + 2×IN immunization regimens overcame this limitation by increasing the frequencies of IFN-γ-secreting T cells and RBD-specific plasma cells in the spleen. Therefore, the combination of intramuscular and intranasal immunization, particularly one dose of intramuscular followed by two intranasal deliveries of the RBD_XBB.1.5_-HR vaccine (1×IM + 2×IN), may elicit both mucosal and systemic immune responses to protect against SARS-CoV-2 variant infections.

### Intranasal RBD_XBB.1.5_-HR vaccine as a booster shot generates enhanced mucosal and systemic immune responses

Messenger RNA (mRNA)-based SARS-CoV-2 vaccines have been extensively deployed globally.^31,32^ However, repeated administration of the same vaccine type may diminish the capacity of heterologous vaccination to elicit enhanced immunity. mRNA vaccines are predominantly administered via the intramuscular route, limiting the potential and utilization of mucosal immunization. Hence, we proceeded to assess the utilization of the intranasal RBD_XBB.1.5_-HR vaccine in a sequential immunization regimen as an additional heterologous booster shot.

**Fig. 5:**
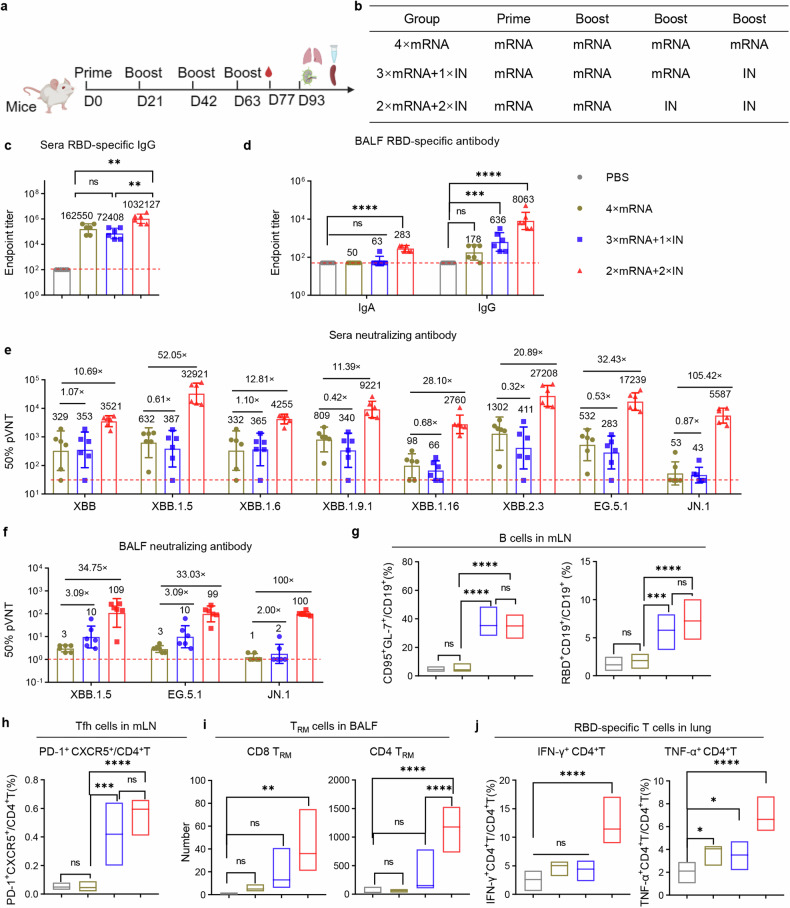
Intranasal RBD_XBB.1.5_-HR vaccine as a heterologous booster shot elicits superior mucosal and systemic immune responses. **a**, **b** NIH mice were immunized three doses of full-length BA.5 spike mRNA vaccine, followed by one dose of homologous injection of mRNA vaccine (4×mRNA), or one heterologous intranasal delivery of RBD_XBB.1.5_-HR vaccine (3×mRNA+1×IN). Mice in another group were received two injections of mRNA vaccine and subsequent two doses of intranasal RBD_XBB.1.5_-HR vaccine (2×mRNA+2×IN) (*n* = 6 mice per group). Endpoint titers of RBD-specific IgG in sera (**c**), and IgA and IgG in BALF samples (**d**). The neutralization against XBB-lineage and JN.1 pseudoviruses in sera (**e**) and BALF samples (**f**). The frequencies of (**g**) GC B, antigen-specific B cells, and (**h**) Tfh cells in mediastinal lymph nodes (*n* = 6 mice each group). **i** The absolute number of CD8^+^ and CD4^+^ T_RM_ cells in BALF samples (*n* = 5 mice each group). **j** The percentages of antigen specific IFN-γ or TNF-α-producing memory CD4^+^ T cells in lung tissue (*n* = 6 mice each group). Data are presented as geometric mean values ± SD in **c–f**. The middle line indicates the median and the box shows the data range in **g–j**. *P* values were conducted by One-way ANOVA analysis followed by Tukey’s multiple comparison post hoc test in **c**, **d** and **g–i**. ****P < 0.0001; ***P < 0.001; **P < 0.01; *P < 0.05; ns not significant

We initially developed an mRNA vaccine encoding the full-length BA.5 spike protein and administered intramuscular immunizations to NIH mice on days 0, 21, and 42, followed by either a homologous fourth-dose mRNA vaccine injection (4×mRNA), or heterologous intranasal delivery of RBD_XBB.1.5_-HR vaccine (3×mRNA+1×IN RBD_XBB.1.5_-HR) on day 63 (Fig. 5a, b). In addition, another group of mice received two doses of the mRNA vaccine followed by two intranasal administrations of the RBD_XBB.1.5_-HR vaccine (2×mRNA+2×IN RBD_XBB.1.5_-HR) (Fig. 5a, b). Notably, a single dose of intranasal RBD_XBB.1.5_-HR vaccine did not appear to induce a stronger systemic humoral immune response, as evidenced by similar levels of sera binding and neutralizing antibodies compared to the 4×mRNA group (Fig. 5c, e). However, two injections of mRNA followed by two intranasal deliveries of RBD_XBB.1.5_-HR (2×mRNA+2×IN RBD_XBB.1.5_-HR) induced a superior systemic humoral immune response, with the highest levels of sera antigen-specific IgG (Fig. 5c) and neutralizing antibodies (Fig. 5e). Compare to homologous mRNA vaccination group (4×mRNA), the GMTs against XBB, XBB.1.5, XBB.1.6, XBB.1.9.1, XBB.1.16, XBB.2.3, EG.5.1 and JN.1 pseudoviruses increased by 10.69-, 52.05-, 12.81-, 11.39-, 28.10-, 20.89-, 32.43- and 105.42-fold, respectively, in the 2×mRNA+2×IN RBD_XBB.1.5_-HR group. In line with the antibody assays, the plasma cells in lung tissue were significantly improved by two intranasal deliveries rather than one single dose of intranasal RBD_XBB.1.5_-HR vaccine (Supplementary Fig. 6). However, we observed enhanced mucosal antibody responses induced by at least one intranasal delivery of the RBD_XBB.1.5_-HR vaccine (Fig. 5d, f).

Local mucosal cellular immune response was also assessed. Consistent with the findings of the respiratory tract antibody assay, intranasal delivery of the RBD_XBB.1.5_-HR vaccine led to the generation of mucosal T_RM_ cells and antigen-specific T cells. Both single and double intranasal administrations of the RBD_XBB.1.5_-HR vaccine induced substantial GC B cells, antigen-specific B cell and Tfh responses within the mediastinal lymph nodes (Fig. 5g, h). Although the number of T_RM_ cells was elevated without statistical significance, it was observed that a single booster dose of the RBD_XBB.1.5_-HR vaccine (3×mRNA+1×IN RBD_XBB.1.5_-HR) might not be sufficient to induce superior mucosal T cellular immune responses compared to homologous mRNA vaccination (4×mRNA) (Fig. 5i, j). In addition, heterologous vaccination involving two prior injections of mRNA followed by two administrations of the RBD_XBB.1.5-_HR vaccine (2×mRNA+2×IN RBD_XBB.1.5_-HR) resulted in the highest counts of CD8^+^ and CD4^+^ T_RM_ cells (Fig. 5i), as well as the highest proportions of IFN-γ-, TNF-α-secreting CD4^+^ T cells (Fig. 5j). These findings suggest that the intranasal RBD_XBB.1.5_-HR vaccine, especially when administered in two doses, represents a promising candidate for boosting to elicit superior mucosal and systemic immune responses in heterologous immunization strategies.

### Intranasal delivery of RBD_XBB.1.5_-HR vaccine confers effective protection against the challenge of live EG.5.1 variant in respiratory tract

**Fig. 6:**
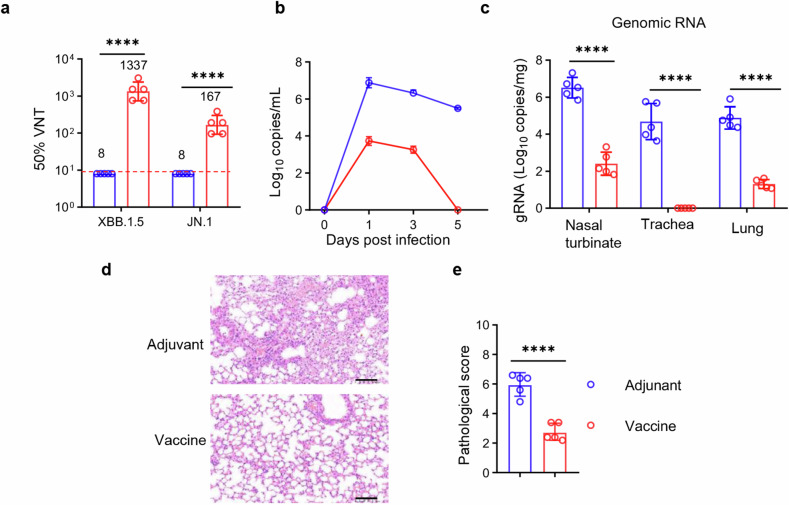
Intranasal RBD_XBB.1.5_-HR vaccine provides protection against the infection of live Omicron EG.5.1 virus. **a** NIH mice were intranasally immunized three times with RBD_XBB.1.5_-HR vaccines. Mice treated with adjuvant were used as controls. Prior to the viral challenge, the sera were collected to determine the neutralizing antibody titers to authentic viruses, including XBB.1.5 and JN.1 variants. **b** On day 21, after the final boost, immunized NIH mice were challenged with 1 × 10^6^ PFU of live SARS-CoV-2 EG.5.1 Omicron viruses via intranasal route (*n* = 5 mice in each group). Changes in viral loads in throat swabs post-SARS-CoV-2 infection were monitored daily. **c** The mice were euthanized on day 5 post infection, and multiple respiratory tissues including nasal turbinates, trachea and lung were collected to detect the levels of gRNA. The representative images of histopathological changes (**d**) and pathological score (**e**) in lung tissue from immunized mice infected with EG.5.1 viruses. Scale bars represent 100 μm in **d**. Data are presented as geometric mean values with SD in **a** and **c**, and as mean values ± SEM in **b** and **e**. *P* values in **a, c** and **e** were conducted by unpaired Student’s *t*-tests. ****P < 0.0001; ***P < 0.001; **P < 0.01; *P < 0.05

In the subsequent experiment, our aim was to assess the effectiveness of the intranasal RBD_XBB.1.5_-HR vaccine against the circulating EG.5.1 virus in vivo. NIH mice were intranasally immunized with three doses of the high-dose RBD_XBB.1.5_-HR vaccine (10 μg per mouse) and subsequently challenged with live EG.5.1 viruses (1 × 10^6^ plaque-forming units (PFU)) via the intranasal route on day 21 after the final immunization. Prior to the viral challenge, serum samples were collected to evaluate the neutralization against authentic viruses, including XBB.1.5 and JN.1 viruses. Consistent with the results of pseudovirus neutralization assays, intranasal delivery of RBD_XBB.1.5_-HR elicited efficient neutralization activities against these circulating variants, with the GMTs for 50% neutralization against XBB.1.5, and JN.1 were 1337 and 167, respectively (Fig. 6a).

After being challenged with live EG.5.1 viruses, the changes in viral loads in throat swabs were monitored daily using a reverse-transcription quantitative polymerase chain reaction (RT–qPCR) assay. Samples from mice administered with adjuvant alone exhibited high levels of viral burden throughout the experimentation period (Fig. 6b). Conversely, intranasal administration of the RBD_XBB.1.5_-HR vaccine markedly reduced viral genomic RNA (gRNA) on day 1 post-infection (dpi), and demonstrated rapid clearance of viruses. By 5 dpi, negligible viral loads were observed in throat swab samples from the group that received the intranasal RBD_XBB.1.5_-HR vaccine.

On day 5 dpi, the mice were then euthanized for tissue collection. In comparison to the administration of adjuvant, intranasal immunization with the RBD_XBB.1.5_-HR vaccine led to significant reductions in viral gRNA levels in nasal turbinates and lung tissues (Fig. 6c). Furthermore, no detectable gRNA was found in the tracheal tissue from the vaccinated group. These findings strongly indicate that the intranasal RBD_XBB.1.5_-HR vaccine offers effective protection against live Omicron viruses in both the upper and lower respiratory tracts.

In addition to the changes in viral loads, the effective protection conferred by the intranasal RBD_XBB.1.5_-HR vaccine also was also associated with significantly mitigated histopathological alterations. In lung tissues from mice treated with PBS, mild pathological changes were evident, including multifocal areas of consolidation, mild thickening of alveolar septa, alveolar congestion, and small patches of inflammation composed of lymphocytes, neutrophils, and macrophages (Fig. 6d, e). In contrast, lung tissues from the group receiving the intranasal RBD_XBB.1.5_-HR vaccine exhibited a normal histological structure, with intact pulmonary alveolar architecture and an absence of apparent inflammation. Thus, the intranasal RBD_XBB.1.5_-HR vaccine effectively protected against the challenge with live EG.5.1 virus in both the upper and lower respiratory tracts.

## Discussion

Given the persistence of various Omicron subvariants and the predominance of intramuscular COVID-19 vaccines, there is an urgent need for next-generation intranasal COVID-19 vaccines. Subunit proteins offer a promising platform for developing these vaccines, and several novel mucosal adjuvants and delivery systems for protein antigens have shown promising results in preclinical studies.^15–20^ Nevertheless, the selection of adjuvants that have already undergone clinical evaluation in humans could significantly accelerate the clinical translation process.

In this study, we evaluated the protective efficacy of a trimeric recombinant protein antigen combined with an MF59-like oil-in-water adjuvant delivered intranasally. Our results demonstrate that intranasal delivery of the adjuvanted RBD_XBB.1.5_-HR, whether as a standalone vaccine or a heterologous booster, can elicit significant and sustained local mucosal and systemic immune responses. The neutralizing antibody responses induced by this intranasal vaccine showed effectiveness against a range of variants, including the recently emerged JN.1, KP.2, and KP.3. Additionally, combining the intranasal route with the intramuscular route resulted in enhanced systemic and mucosal protective immunity. Notably, the intranasal RBD_XBB.1.5_-HR vaccine provided efficient protection against authentic EG.5.1 virus challenges in the respiratory tract. However, while our study confirms the efficacy of MF59-like as an adjuvant for SARS-CoV-2 intranasal vaccines, the specific immune mechanisms through which the MF59-like oil-in-water adjuvant induces robust mucosal immunity have not been thoroughly explored and warrant further investigation. Nonetheless, these findings suggest a viable strategy for selecting clinically proven, safe, and effective intranasal vaccine adjuvants, thereby advancing their clinical application.

The antigenic composition of a vaccine is a crucial determinant of its effectiveness, particularly in the context of COVID-19 vaccines targeting various variants with enhanced immune evasion capabilities. However, most current vaccines utilize antigens derived from previously circulating strains. Consequently, for the next generation of intranasal vaccines, it is imperative to select antigens derived from recently emerged variants that exhibit strong immunogenicity capable of eliciting cross-neutralizing responses. Moreover, repeated exposure to prior antigens tends to bias immune responses toward earlier lineage variants, thereby diminishing the immune efficacy against more recent Omicron subvariants.^33–36^ In light of these considerations, the Food and Drug Administration (FDA) has proposed the utilization of a component from the XBB descendant lineage as the vaccine antigen, leading to the marketing authorization of XBB.1.5 monovalent mRNA vaccines by Moderna and Pfizer/BioNTech.^36^ Our previous studies have also demonstrated that immunization with an antigen derived from the XBB.1.5 variant can elicit a broad spectrum of neutralizing capacities against universal Omicron subvariants compared to spike proteins from other Omicron subvariants.^37^ However, to date, there has been no development of a subunit protein-based intranasal vaccine targeting the XBB lineages. Herein, we demonstrate the intranasal delivery of XBB.1.5 RBD-derived trimeric protein antigen formulated with adjuvant induces potent neutralizing activities against XBB-lineage subvariants. Even against the most dominant circulating variant, JN.1,^7,8,38^ the neutralizing antibody induced by the intranasal RBD_XBB.1.5_-HR vaccine remains at a high level, as evidenced by the GMTs of 50% neutralization being 6150.

The hybrid immune landscape within populations, influenced by various factors such as the types of administered vaccines (mRNA, inactivated virus, adenovirus), the sequences of encountered variants (pre-Omicron, Omicron, XBB-lineage), and the temporal intervals between vaccination and infection, presents an increasingly complex challenge for the development of next-generation vaccine boosters.^39,40^ Therefore, exploring the potential of intranasal vaccines as booster shots in heterologous vaccination regimens holds significant importance. Although Moderna and Pfizer/BioNTech have updated the sequences of their mRNA vaccines to incorporate XBB.1.5 spike proteins for booster shots,^36^ considering the widespread use of mRNA-based COVID-19 vaccines globally,^31,32,41^ opting for heterologous vaccination with an alternative vaccine platform and incorporating mucosal delivery may offer enhanced systemic and mucosal immune responses.^42^ Our findings in the present study demonstrate that superior antibody responses can be achieved through heterologous immunization with two intranasal administrations of the RBD_XBB.1.5_-HR vaccine following mRNA injections, accompanied by robust local mucosal immune responses. We observed that a single dose of intranasal RBD_XBB.1.5_-HR vaccine did not significantly augment the levels of neutralizing antibodies in sera compared to homologous vaccination. However, it did indeed provide additional mucosal protective immunity.

One drawback of intranasal delivery is its tendency to evoke comparatively lower systemic and cellular immune responses compared to intramuscular administration.^10,12,43^ Consistent with prior research, it has been shown that three administrations of an intranasal vaccine inadequately stimulate antigen-specific T cells and memory B cells in spleen tissue. However, a strategy combining both intramuscular and intranasal vaccination routes appears to offer a viable solution to this challenge.^44^ Specifically, the inclusion of at least one intramuscular injection prior to intranasal administration can elicit a systemic cellular immune response (e.g., 2×IM + 1×IN, 1×IM + 2×IN). Given that a significant portion of the population has already received at least one dose of intramuscular vaccine, this concern may not be of substantial concern. In addition, our investigation revealed that two intranasal administrations of the RBD_XBB.1.5_-HR vaccine following a single intramuscular injection (1×IM + 2×IN) resulted in the highest levels of systemic and mucosal antibodies compared to other groups (e.g., 2×IM + 1×IN, 3×IN). While at least one intranasal delivery can stimulate a local mucosal response, as evidenced by the generation of cellular immune responses in the lung and germinal center reactions in mediastinal lymph nodes. These findings suggest that for optimal induction of humoral and cellular immune responses in both systemic and local mucosal immunity, adjuvanted RBD_XBB.1.5_-HR vaccines should be administered intranasally at least twice. In summary, our study systematically assessed the immunogenicity of the trimeric antigen RBD_XBB.1.5_-HR protein formulated with MF59-like oil-in-water as a standard intranasal vaccine or heterologous booster shot. Additionally, we explored the effects of various immunization regimens on vaccine protective efficacy. These findings provide an important theoretical foundation for expediting the development of an intranasal vaccine that can be swiftly translated into clinical practice.

## Materials and methods

### Vaccine preparation

In this study, the RBD derived from the SARS-CoV-2 XBB.1.5 variant was directly fused with HR1 and HR2 in the spike protein S2 subunit. Additionally, the C538 residue site within the RBD was replaced with serine to avoid the formation of inter-chain disulfide bonds. Leveraging the self-assembly property of the HR sequence, we successfully generated the trimeric antigen RBD_XBB.1.5_-HR protein. The Bac-to-Bac baculovirus expression system (Invitrogen) was employed to produce the recombinant trimeric protein. Briefly, the gene was expanded and inserted into the pFastBac1 vector, which was subsequently transformed into *E.* *coli* DH10b cells for cloning. The resulting recombinant bacmids were subsequently transfected into Sf9 insect cells to produce the antigen protein. Following expression, the protein underwent further purification and characterization. Subsequently, the antigen protein RBD_XBB.1.5_-HR was mixed with adjuvant (MF59-like oil-in-water) at a 1:1 volume ratio to prepare the recombinant vaccine.

### Animal vaccination

The experiments involving mice and rats were conducted in accordance with the guidelines established by the Institutional Animal Care and Use Committee of Sichuan University (Chengdu, Sichuan, China) (Ethical approval number: 20230227017). Specific pathogen-free (SPF) female National Institute of Health (NIH) Swiss mice aged 6-8 weeks were procured from Beijing Vital River Laboratory Animal Technologies Co., Ltd (China). All animals were housed in a specific pathogen-free (SPF) facility at the State Key Laboratory of Biotherapy, with a temperature range of 21–25 °C, humidity between 30–70%, and a 12-hour light/dark cycle.

They were housed in a SPF animal facility located in the animal center of the State Key Laboratory of Biotherapy and acclimatized for a period of 1 week prior to the commencement of the experiment. The NIH mice were randomly divided into groups and intranasally administered with 50 μL of PBS, RBD_XBB.1.5_-HR, MF59-like, or a vaccine formulation containing RBD_XBB.1.5_-HR with adjuvant on days 0, 21, and 42. Mice in the low-dose group received 5 μg of the vaccine, while those in the high-dose group received 10 μg. Rats were immunized with either 200 μL of PBS or 40 μg of RBD_XBB.1.5_-HR combined with adjuvant, following the same prime-boost regimen with a 21-day interval. Blood samples were collected from mice and rats at 2, 5, or 8 weeks after the initial immunization or 6 months after the completion of immunization.

To evaluate the immune response resulting from the combination of intranasal (IN) and intramuscular (IM) immunization, NIH mice were administered three doses of 10 μg RBD_XBB.1.5_-HR combined with adjuvant via either IM or IN injection on days 0, 21, and 42. Mice in the 1×IM + 2×IN group received one dose via IM injection followed by two doses via IN injection. Mice in the 2×IM + 1×IN group were given two doses via IM injection followed by one dose via IN injection. Mice in the 3×IN group received three doses of the vaccine exclusively via IN delivery.

### Enzyme-linked immunosorbent assay (ELISA)

ELISA was employed to measure RBD-specific IgG and IgA levels. In brief, RBD_XBB.1.5_-HR (1 μg/mL) was fixed onto 96-well plates (NUNC-MaxiSorp, Thermo Fisher Scientific) through overnight incubation at 4 °C or for 2 h at room temperature. After being washed three times with PBST (PBS with 0.1% Tween 20), each well was loaded with 100 μL of diluted sera or BALF. After incubating at 37 °C for 1 hour, the plates were washed again with PBST. Subsequently, 100 μL of HRP-conjugated anti-mouse IgA, IgG, IgG1, IgG3, IgG2a, IgG2b, IgG2c, or anti-rat IgG antibodies (1:10,000) was added to each well and incubated at 37 °C for 1 hour. After five washes, 100 μL of TMB (3,3′,5,5′-tetramethyl biphenyl diamine) substrate was added to each well for 10-minute reaction at room temperature, 50 μL of stop buffer (H_2_SO_4_) was added to terminate the color development, and the absorbance at 450 nm was measured with microplate reader (Spectramax ABS, Molecular Devices).

### SARS-CoV-2 pseudovirus neutralization assay

The pseudovirus neutralization assay is a widely used method to assess the presence of neutralizing antibodies in serum or BALF. Genomeditech provided us with XBB, XBB.1.5, XBB.1.6, XBB.1.9.1, XBB.1.16, XBB.2.3, EG.5.1, JN.1, KP.2 and KP.3 pseudoviruses. Serum or BALF samples were initially heat-inactivated in a 56 °C water bath for 30 minutes, then diluted in a 3-fold gradient in 96-well plates (Cat: WHB-96-03, Shanghai Wohong Biotechnology Co., Ltd.). Subsequently, diluted pseudovirus (50 μL/well) was added to the plate and incubated at 37 °C for 1 hour. Following this, 293 T/ACE2 cells (1.5 × 10^4^/well) were added to the plate and cultured at 37 °C with 0.5% CO_2_ for two days to allow for luciferase expression. On the final day, the supernatant was removed, 100 µL luciferase substrate was added to each well, then luminescence was read using a multi-mode microplate reader (PerkinElmer, USA) with Kaleido 3.0 software.

### Enzyme linked immunospot assay (ELISpot)

For the detection of IFN-γ-secreting lymphocytes in the spleen, 96-well ELISpot plates (Cat: 3420-4APT-2, MABTECH) were washed four times with PBS and then closed with 1640 complete medium (10% FBS, 100 µL/well) at 37 °C for 1 hour. After gently removing the supernatant, isolated lymphocytes (3 × 10^5^/well) were added to each well. The cells in the plates were stimulated overnight with pools of XBB.1.5 spike protein peptides in an incubator (37 °C, 5% CO_2_). After washing off the cells with PBS, the plates were incubated with detection 1 μg/mL 7-B6-1-biotin antibodies at room temperature for two hours. Following five washes with PBS, the plates were incubated with Streptavidin-ALP (1:1000) for 1 hour at room temperature. The plates were washed five times using PBS, and then the substrate solution (BCIP/NBT - plus, 100 μL/well) was added to form spots.

For the detection of IgG^+^ or IgA^+^ antibody-secreting cells (ASCs) in the lung and spleen, multiscreen 96-well filtration plates (Millipore, REF: MSIPS4W10) were initially washed and then coated with RBD_XBB.1.5_-HR (3 μg/mL) overnight at 4 °C. After washing and blocking, 30,000 lymphocytes from the spleen or lung were added to each well and cultured at 37 °C. After 16 h, the cells in the wells were gently washed out. Subsequently, 100 μL of HRP-conjugated IgG or IgA secondary antibodies were added to each well and incubated at room temperature for 2 h. After washing with PBS, 100 μL of TMB substrate for HRP (code: 3651-10, MABTECH) was added to each well to enable spot formation. After incubation at room temperature for 10 minutes, the plates were rinsed with water and dried. Finally, the spots were read using an AID ELISpot Reader.

### Flow cytometry

For the detection of tissue resident memory (T_RM_) cells in bronchoalveolar lavage fluid, cells from mice were collected 30 days after the final immunization and stained with PerCP/Cyanine5.5-conjugated anti-mouse CD3 (BioLegend, Cat#100218), Brilliant Violet 510™-conjugated anti-mouse CD8 (BioLegend, Cat#100751), Brilliant Violet 421™-conjugated anti-mouse CD4 (BioLegend, Cat#100438), PE-conjugated anti-mouse CD69 (BioLegend, Cat# 164204), PE/Cyanine7-conjugated anti-mouse CD44 (BioLegend, Cat# 103030), and APC-conjugated anti-mouse CD103 (BioLegend, Cat#121414) antibodies.

For the assay of T follicular helper (Tfh) cells in mediastinal lymph nodes (mLN), the cells were labeled with the following antibodies: PerCP/Cyanine5.5-conjugated anti-mouse CD3, Brilliant Violet 421-conjugated anti-mouse CD4, PE/Cyanine7 anti-mouse/human B220 (BioLegend, Cat# 103222), APC anti-mouse CD185 (CXCR5, BioLegend, Cat# 145506), and FITC anti-mouse CD279 (PD-1, BioLegend, Cat# 135214). For the detection of germinal centers (GCs) and RBD-specific B cells, cells in mLN were treated with Biotinylated RBD protein (1 μg/mL, SPD-C82Q3, ACROBiosystems) for 30 minutes at room temperature. After washing with PBS, cells were stained with PerCP/Cyanine5.5-conjugated anti-mouse CD3, Pacific Blue™ anti-mouse CD19 (BioLegend, Cat# 152416), APC anti-mouse CD95 (BioLegend, Cat# 152604), Alexa Fluor® 647 anti-mouse/human GL7 (BioLegend, Cat# 144606), and streptavidin-conjugated PE (BioLegend, Cat# 405204).

Lung tissues were aseptically collected and minced. Subsequently, the tissues were enzymatically digested in a buffer comprising DMEM medium (Gibco, USA) supplemented with 1 mg/mL collagenase I (Gibco, USA), 0.5 mg/mL collagenase IV (Gibco), and 40 U/mL DNase I (KeyGen biotech) at 37 °C for one hour. Following digestion, the tissue homogenate was filtered through a 70-mesh screen, subjected to red blood cell lysis, then washed by PBS to obtain single-cell suspensions. Lymphocytes from the spleen were isolated using mouse lymphocyte isolation solution. T cells from lung tissue or lymphocytes from the spleen were cultured overnight in 1640 complete medium with a spike peptide pool (1 μg/mL). Brefeldin A (BFA, Invitrogen, Cat# 00-4506-51) was added to the culture four hours before cell collection to block intracellular cytokine secretion. Subsequently, the cells were stained with PerCP/Cyanine5.5-conjugated anti-mouse CD3, FITC anti-mouse CD8 (BioLegend, Cat# 100705), and APC anti-mouse CD4 (BioLegend, Cat# 100412) antibodies at 4 °C. Afterward, the cells were fixed and treated with anti-mouse antibodies: PE/Cyanine7 IFN-γ (BioLegend, Cat# 505826) and Brilliant Violet 510™ TNF-α (BioLegend, Cat# 506339).

In spleen cells, the RBD^+^ plasma cells were detected by first treating the cells with Biotinylated RBD protein for 30 minutes, followed by washing with PBS. The cells were then stained with Brilliant Violet 510™ anti-mouse CD4 (BioLegend, Cat# 116025), PE/Cyanine5 anti-mouse CD19 (BioLegend, Cat# 115510), APC anti-mouse/human GL7 (BioLegend, Cat# 144618), Brilliant Violet 421™ anti-mouse C138 (BioLegend, Cat# 144618), FITC anti-mouse IgD (BioLegend, Cat# 405704), and streptavidin-conjugated PE for the detection of RBD^+^ plasma cells.

Nine months after the initial vaccination, memory B cells in the lung, mLN, and spleen were detected and stained using the following antibodies: FITC-anti-mouse CD45R (BioLegend, Cat#103206), PerCP/Cyanine5.5-anti-mouse CD19 (BioLegend, Cat#152406), PE/Cy7-anti-mouse CD38 (BioLegend, Cat#102718), Brilliant Violet 421™-anti-mouse IgD (BioLegend, Cat#405725), APC-anti-mouse GL-7 (BioLegend, Cat#144618), and streptavidin-conjugated PE. All cell samples were analyzed using the NovoCyte Flow Cytometer (ACEA Biosciences) using NovoExpress 1.4.1 software.

### Challenge of mice with live Omicron EG.5.1 viruses

NIH mice (aged 6-8 weeks) from the vaccine group, were intranasally administered three doses of 10 μg adjuvanted-RBD_XBB.1.5_-HR on days 0, 21, and 42, while mice in the control group received three doses of adjuvant. Twenty-one days after the final immunization, all mice were intranasally challenged with live EG.5.1 virus (1 × 10^6^ PFU/mice). Viral loads in throat swabs were monitored on days 1, 3, and 5 post-challenge. Five days after the challenge, mice were euthanized, and lung tissues were gathered for viral load assays and histological examination. Hematoxylin and eosin staining (H&E) were employed to assess pathological changes in lung tissue. Viral genomic RNA (gRNA) in lung tissue and throat swabs was detected using reverse-transcription quantitative polymerase chain reaction (RT-qPCR). The primer sequences used were 5’-GACCCCAAAATCAGCGAAAT-3’ (forward) and 5’-TCTGGTTACTGCCAGTTGAATCTG-3’ (reverse), with the probe sequence being 5’-FAM-ACNGCCGCATTACGTTTGGTGGACC-BHQ1-3’.

All the procedures in which mice were challenged with live EG.5.1 viruses were comprehensively reviewed and approved by the Institutional Animal Care and Use Committee of the Institute of Medical Biology, Chinese Academy of Medical Sciences, and were carried out in the ABSL-4 facility of the Kunming National High-level Biosafety Primate Research Center (Ethical approval number: DWSP202312014).

### Statistics

The data are presented as geometric mean values ± standard deviation (SD), median and with the data range, or mean values ± standard error of mean (SEM) as indicated in each figure legend and statistical analyses were performed using Prism 9.0 (GraphPad software). *P* values were calculated using Student’s *t*-tests for comparisons between two groups and One-way ANOVA for multiple group comparisons. ****P < 0.0001; ***P < 0.001; **P < 0.01; *P < 0.05; ns, not significant.

## Supplementary information

Original image of ELISPOT

Supplementary Materials

## Data Availability

All data that support the findings of this study are available in the main text and supplement information. All other relevant data are available from the lead contact upon reasonable request.
