# Supplementary figures and images for: Intranasal delivery of a subunit protein vaccine provides protective immunity against JN.1 and XBB-lineage variants
Source: Signal Transduct Target Ther. 2024 Nov 20;9:311. doi: 10.1038/s41392-024-02025-6 (PMC11577066; doi:10.1038/s41392-024-02025-6)

## Slide 1
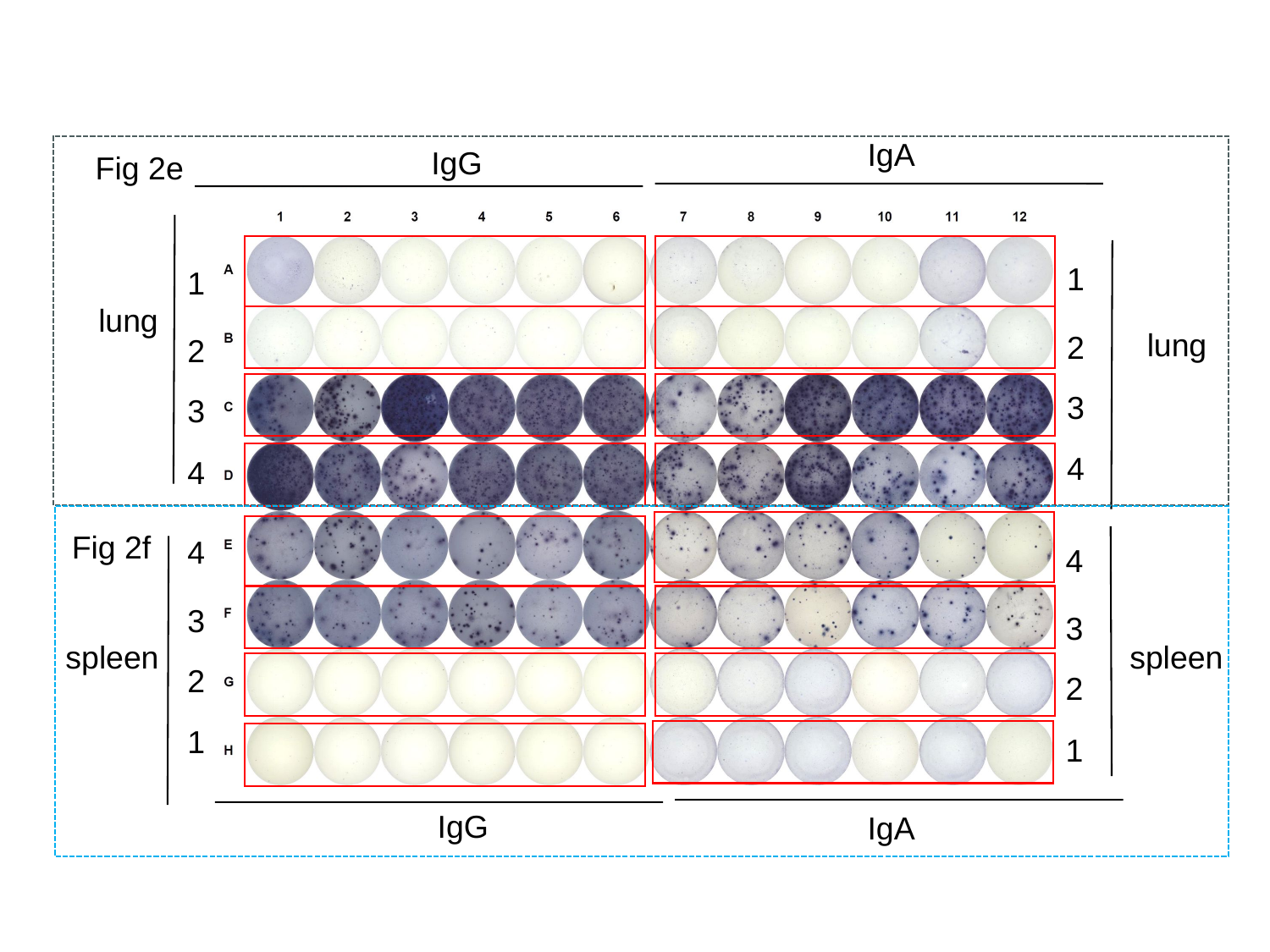

IgA
IgG
Fig 2e
1
1
lung
lung
2
2
3
3
4
4
Fig 2f
4
4
3
3
spleen
spleen
2
2
1
1
IgG
IgA

## Slide 2
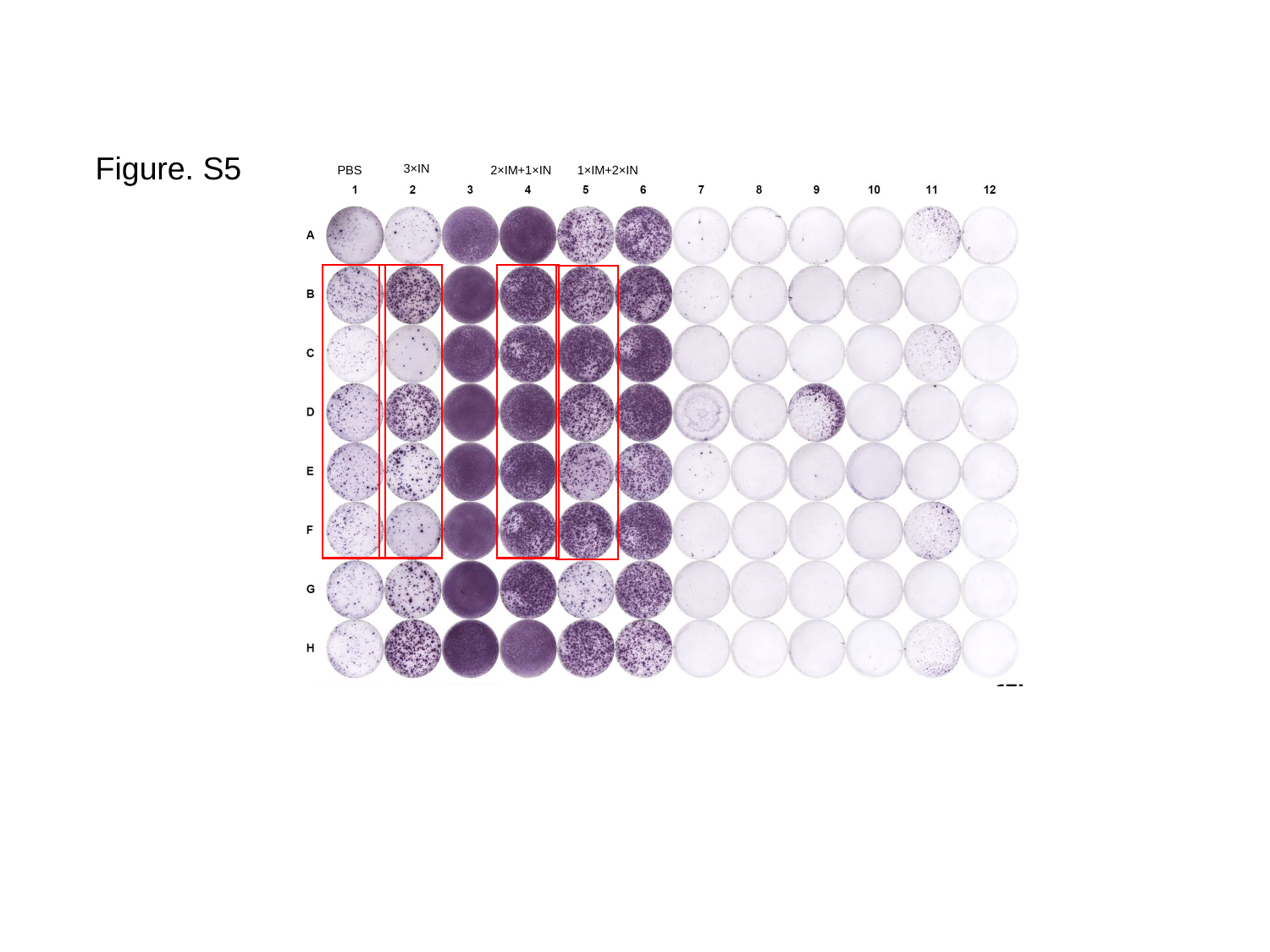

Figure. S5
3×IN
2×IM+1×IN
1×IM+2×IN
PBS

Supplement: Supplementary file 1 — Original image of ELISPOT [file 41392_2024_2025_MOESM1_ESM.ppt]
