# Supplementary material for: Intranasal delivery of a subunit protein vaccine provides protective immunity against JN.1 and XBB-lineage variants
Source: Signal Transduct Target Ther. 2024 Nov 20;9:311. doi: 10.1038/s41392-024-02025-6 (PMC11577066; doi:10.1038/s41392-024-02025-6)
Supplement: Supplementary file 2 — Supplementary Materials [file 41392_2024_2025_MOESM2_ESM.docx]

Supplementary Materials for

Intranasal delivery of a subunit protein vaccine provides protective immunity against JN.1 and XBB-lineage variants

Hong Lei^1^†, Weiqi Hong^1^†, Jingyun Yang^1^†, Cai He^1^†, Yanan Zhou^2^†,Yu Zhang^1^, Aqu Alu^1^, Jie Shi^1^, Jian Liu^1^, Furong qin^1^, Danyi Ao^1^, Xiya Huang^1^, Zimin Chen^1^, Hao Yang^2^, Yun Yang^2^, Wenhai Yu^2^, Cong Tang^2^, Junbin Wang^2^, Bai Li^2^, Qing Huang^2^, Hongbo Hu^1^, Wei Cheng^1^, Haohao Dong^1^, Jian Lei^1^, Lu Chen^1^, Xikun Zhou^1^, Jiong Li^1^, Li Yang^1^, Zhenling Wang^1^, Wei Wang^1^, Guobo Shen^1^, Jinliang Yang^1^, Zhiwei Zhao^1^, Xiangrong Song^1^, Guangwen Lu^1^, Qiangming Sun^2^*, Youchun Wang^2^*, Shuaiyao Lu^2^*, Xiawei Wei^1^*

Correspondence to: xiaweiwei@scu.edu.cn

**This PDF file includes:**

Figures. S1 to S6

Figure. S1.


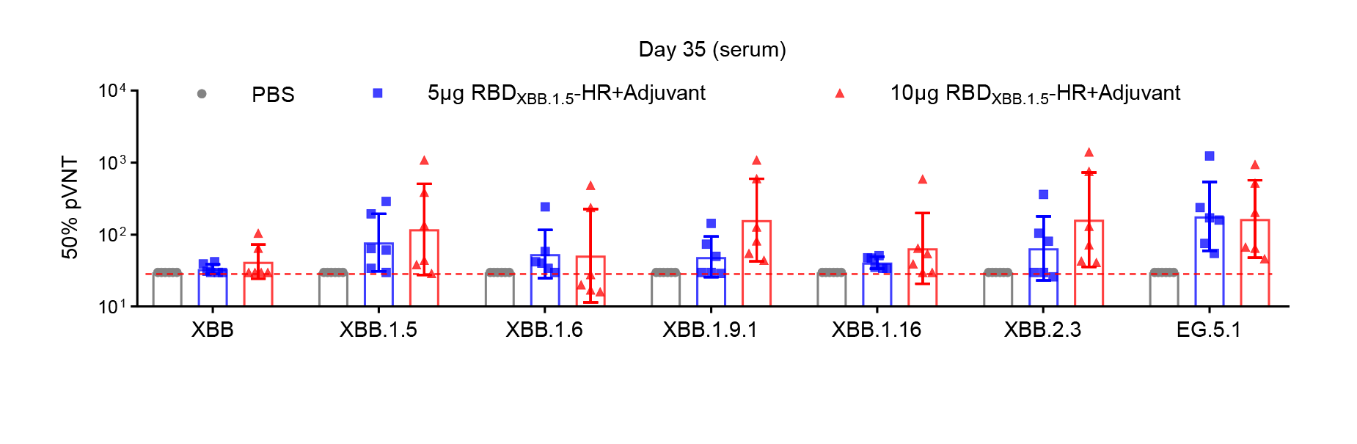


Supplemental Figure 1. Neutralizing antibodies against pseudoviruses in mice sera collected on day 35 (n=6 mice per group). Data are presented as geometric mean values ± SD.

Figure. S2.


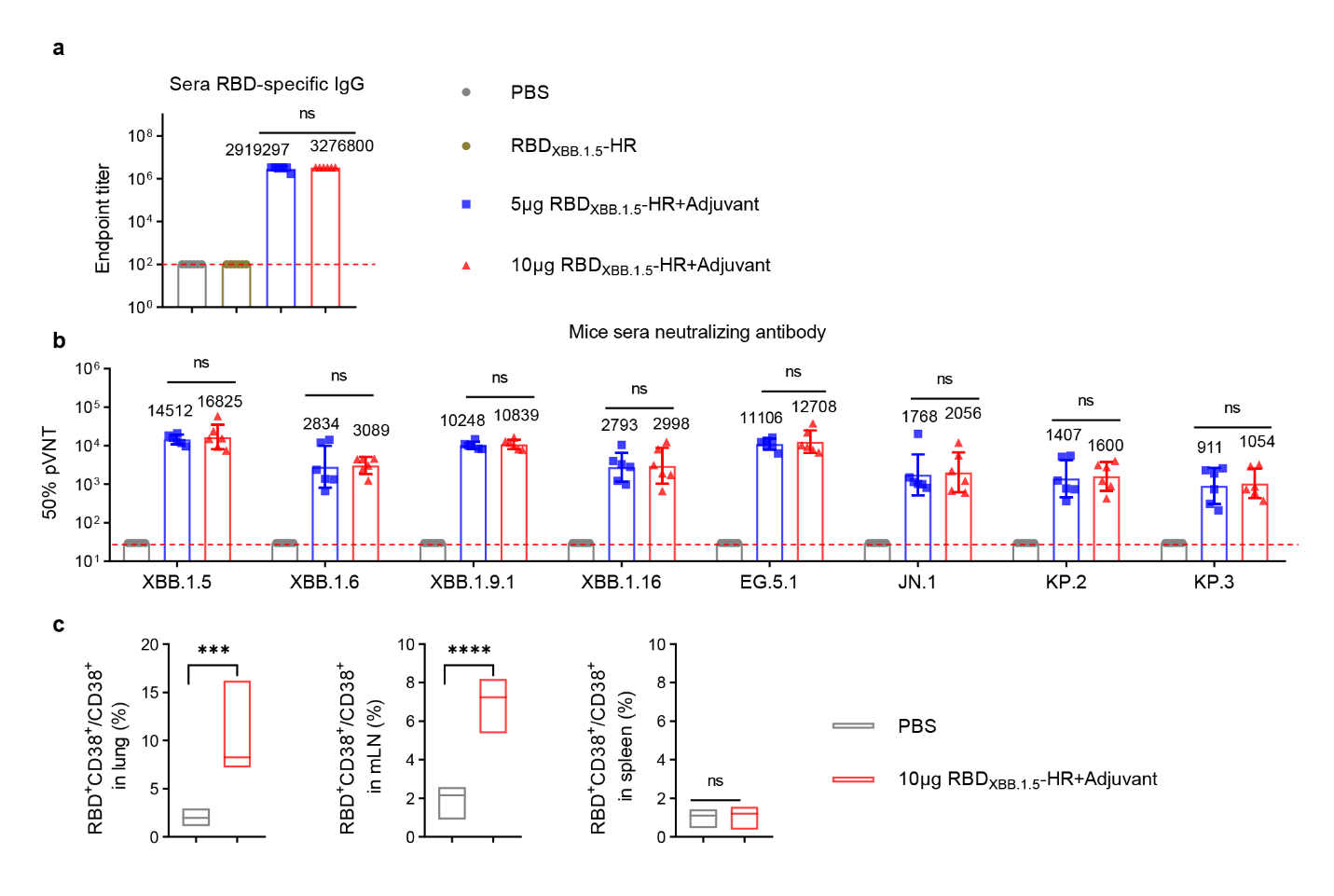


Supplemental Figure 2. Intranasal delivery of adjuvanted-RBD_XBB.1.5_-HR vaccine provides long-term protective immunity. a-b, Female NIH mice were intranasally vaccinated three times with adjuvanted RBD_XBB.1.5_-HR protein. Serum samples were collected six months after the final immunization to assess long-lasting antibody responses, measuring binding (a) and neutralizing antibodies (b) (n=6 mice per group). c, Mice from groups receiving PBS or a high dose of vaccine were sacrificed nine months post-final vaccination. The percentages of memory B cells (MBCs) in lung, lymph nodes and spleen tissues were assessed. Data are presented as geometric mean values with SD for a-b, and the line represents the median and the box shows data range in c. P values in c were conducted by Unpaired Student’s t-tests.

Figure. S3.


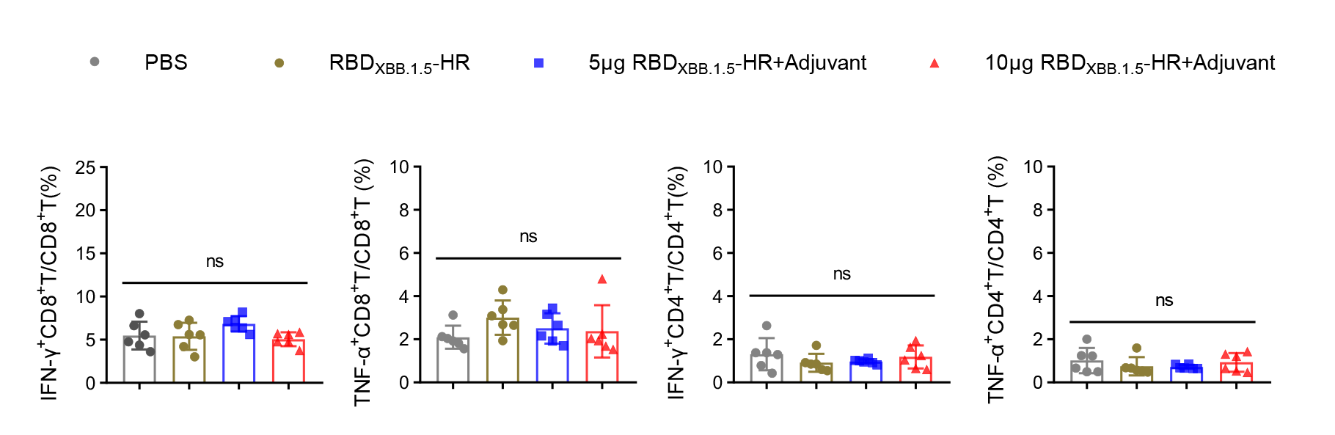


Supplemental Figure 3. The percentages of IFN-γ or TNF-α-secreting CD8^+^ and CD4^+^ T cells in lung tissue in the absence of stimulation of peptide pools for SARS-CoV-2 XBB.1.5 spike (n=6 mice each group). Data are presented as mean with SEM. P values were conducted by One-way ANOVA analysis followed by Tukey’s multiple comparisons test. ns, not significant.

Figure. S4.


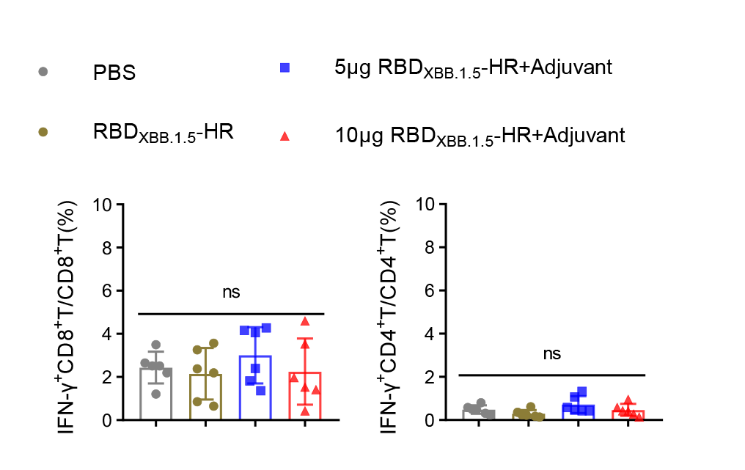


Supplemental Figure 4. The percentages of antigen specific IFN-γ-producing memory CD8^+^ and CD4^+^ T cells in spleen tissues from mice intranasally immunized RBD_XBB.1.5_-HR vaccine (n=6 mice each group). Data are presented as mean values ± SEM. P values were conducted by One-way ANOVA analysis followed by Tukey’s multiple comparison post hoc test. ns, not significant.

Figure. S5.


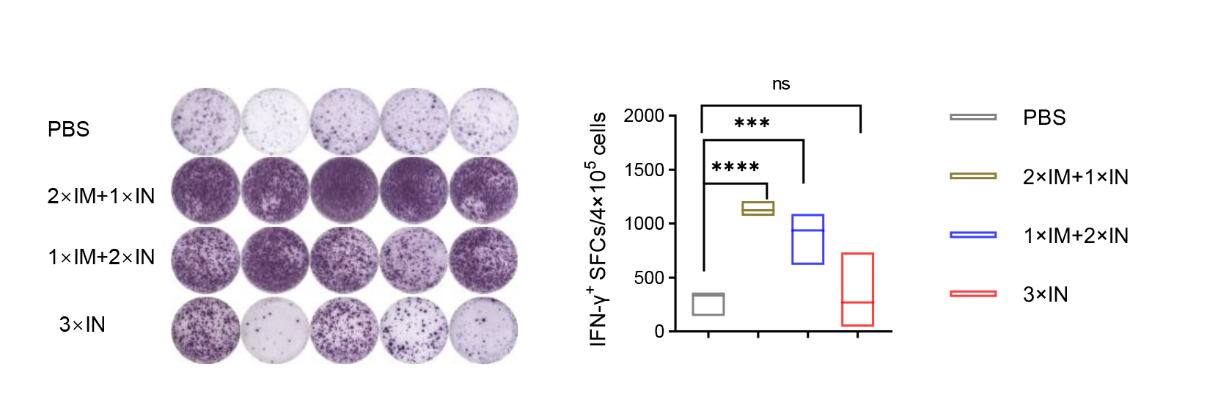


Supplemental Figure 5. The representative images (left) and quantitative analysis (right) of IFN-γ-secreting lymphocytes in spleen after stimulation with peptides pools of XBB.1.5 spike proteins (n=5 mice per group). The middle line indicates the median and the box shows the data range. P values were conducted by One-way ANOVA analysis followed by Tukey’s multiple comparison post hoc test. ****P < 0.0001; ***P < 0.001; ns, not significant.

Figure. S6.


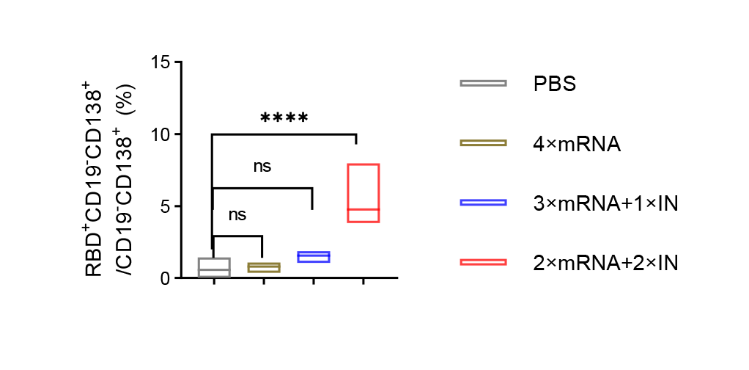


Supplemental Figure 6. The percentages of antigen-specific plasma cells (RBD^+^CD19^-^CD138^+^) in lung tissues from mice with heterologous or homologous vaccinations. The middle line indicates the median and the box shows the data range. P values were conducted by One-way ANOVA analysis followed by Tukey’s multiple comparison post hoc test. ****P < 0.0001; ns, not significant.
